# Prospectively Quantifying the Propensity for Atrial Fibrillation: A Mechanistic Formulation

**DOI:** 10.1371/journal.pone.0118746

**Published:** 2015-03-13

**Authors:** Richard T. Carrick, Oliver R. J. Bates, Bryce E. Benson, Nicole Habel, Jason H. T. Bates, Peter S. Spector

**Affiliations:** 1 Department of Bioengineering, University of Vermont College of Engineering and Mathematical Sciences, Burlington, Vermont, United States of America; 2 Department of Biomedical Engineering, Boston University College of Engineering, Boston, Massachusetts, United States of America; 3 Department of Medicine—Cardiology, University of Vermont College of Medicine, Burlington, Vermont, United States of America; 4 Department of Medicine—Pulmonary Disease and Critical Care Medicine, University of Vermont College of Medicine, Burlington, Vermont, United States of America; Georgia State University, UNITED STATES

## Abstract

The goal of this study was to determine quantitative relationships between electrophysiologic parameters and the propensity of cardiac tissue to undergo atrial fibrillation. We used a computational model to simulate episodes of fibrillation, which we then characterized in terms of both their duration and the population dynamics of the electrical waves which drove them. Monte Carlo sampling revealed that episode durations followed an exponential decay distribution and wave population sizes followed a normal distribution. Half-lives of reentrant episodes increased exponentially with either increasing tissue area to boundary length ratio (*A/BL*) or decreasing action potential duration (*APD*), resistance (*R*) or capacitance (*C*). We found that the qualitative form of fibrillatory activity (e.g., multi-wavelet reentry (MWR) vs. rotors) was dependent on the ratio of resistance and capacitance to *APD*; MWR was reliably produced below a ratio of 0.18. We found that a composite of these electrophysiologic parameters, which we term the fibrillogenicity index (Fb = A/(BL*APD*R*C)), reliably predicted the duration of MWR episodes (r^2^ = 0.93). Given that some of the quantities comprising *Fb* are amenable to manipulation (via either pharmacologic treatment or catheter ablation), these findings provide a theoretical basis for the development of titrated therapies of atrial fibrillation.

## Introduction

Atrial fibrillation (AF) is the most prevalent cardiac arrhythmia in the United States, affecting an estimated 2.66 million adults as of 2010 [1],[2]. Unfortunately, despite the magnitude of its impact on the healthcare system [3], current treatments have proven inadequate, particularly in patients with longer lasting AF [4],[5],[6]. At least in part, this is due to our inability to tailor therapies to the specific electrical abnormalities of individual patients’ hearts. Instead, the severity of AF is categorized in a binary fashion based upon whether episodes of AF last less than or greater than 7 days [7], and ablation strategy is selected based upon this classification (i.e. pulmonary vein encircling alone vs. encircling plus additional atrial ablation) [4],[8]. Because the duration of AF episodes reflects the degree of electrical derangement in the heart, and this property exists along a continuum [9], it is therefore not surprising that basing therapy on a binary system often leads to unsatisfactory results.

It seems reasonable to suppose that better outcomes could be achieved by basing therapy on a more comprehensive assessment of atrial electrophysiology which accounts for the unique characteristics of individual patients’ hearts. Indeed, the relationship between atrial properties and the propensity to fibrillate has long been a subject of intense investigation. As early as 1912, investigators recognized that a “critical mass” of tissue is required to support fibrillation [10],[11],[12]. In addition, it has long been appreciated that shortened refractory period and slowed conduction velocity are pro-fibrillatory [13],[14],[15]. Nevertheless, the precise manner in which these factors determine the atria’s propensity to fibrillate remains poorly understood, and thus the degree to which they should be manipulated in the treatment of AF is unclear.

Accordingly, the purpose of this study was to quantify how electrophysiologic parameters determine the propensity of atrial tissue to support fibrillation. We pursued this goal with the aid of a previously described computational model which allowed us to study the behavior of multi-wavelet reentry (MWR), a chaotic propagation pattern which has long been considered one of the key mechanisms of AF [16],[17],[18]. In a previous paper, we introduced the idea of viewing MWR from a population dynamics perspective [19]. The wave population varies as a function of wave births and deaths and MWR terminates when the population reaches zero. We hypothesized that while the destinies of individual waves cannot be easily anticipated, the effect of parameter changes (e.g., changes in area, boundary length and wavelength) on average wave behavior (i.e. AF duration) can be quantitatively predicted. Further, we propose that changes to these parameters are pro- or anti-fibrillatory based upon their relative impact on the probabilities of wave births and deaths.

## Methods

We performed a series of numerical experiments with a previously described computational model of cardiac electrical propagation [12],[19],[20]. Model code was written in C++ and simulations were run on the Vermont Advanced Computing Core (see www.uvm.edu/∼vacc/ for specifications). The model consists of a rectangular grid of excitable “cells” (each representing a roughly 1 mm^2^ group of cells) having a combined area of *A* and an outside boundary length of *BL*. The cells are connected to each other by electrically conducting pathways of resistance *R* via von Neumann neighborhoods (up, down, right and left). Because relative rather than absolute voltages guide the model’s behavior, each cell had a voltage which varied on a continuous scale between a minimum, *V*_*min*_ = 0, representing the resting or polarized state, and a maximum, *V*_*max*_ = 1, representing the fully depolarized state. Pairs of connected cells exchange charge at a rate determined by their voltage differences and *R*. The accumulated charge in a cell determines its voltage in inverse proportion to its capacitance *C*. When the voltage of a cell reaches a set threshold *V*_*min*_<*V*_*thresh*_<*V*_*max*_ that cell’s voltage ascends rapidly to *V*_*max*_, and is then refractory for the duration of the action potential, *APD*. In this way, the depolarization of one cell spreads to its neighbors to generate a propagating action potential. The model time step was scaled relative to the length of typical action potentials such that it represented approximately 1ms. A degree of heterogeneity was also introduced by randomizing cellular *APD*s around mean values using a uniform distribution of +/−10ms.

Reentrant activity was initiated in the model via high-frequency burst pacing (100Hz for 1s) from a virtual electrode placed over a randomly chosen cell. Due to the chaotic nature of MWR, even small changes in the position from which reentry was induced could result in large differences in the state of the model’s activity at a given time step; thus, induction from each electrode location produced a unique episode of MWR. The qualitative nature of reentry depended on the values of the model parameters. Reentrant patterns were visually categorized by an observer blinded to the model parameters into four distinct groups: pure MWR, MWR with occasional rotor formation, rotors with occasional bouts of MWR, or rotors only (see S1 Video for representative examples of these reentrant phenotypes).

**Fig 1:**
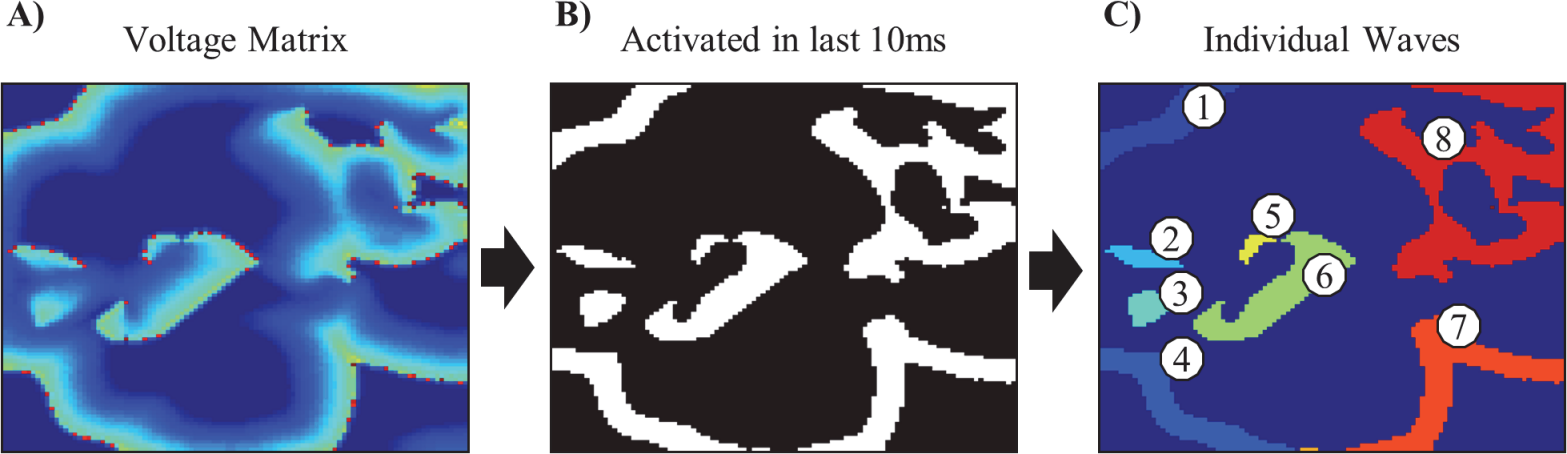
Wave Identification Algorithm. *A)* – an example of MWR in the computational model, *B)*—the cells that became depolarized within the previous 10ms (shown in white), *C)* – the eight distinct groups of contiguous depolarized cells which constitute the individual waves.

**Fig 2:**
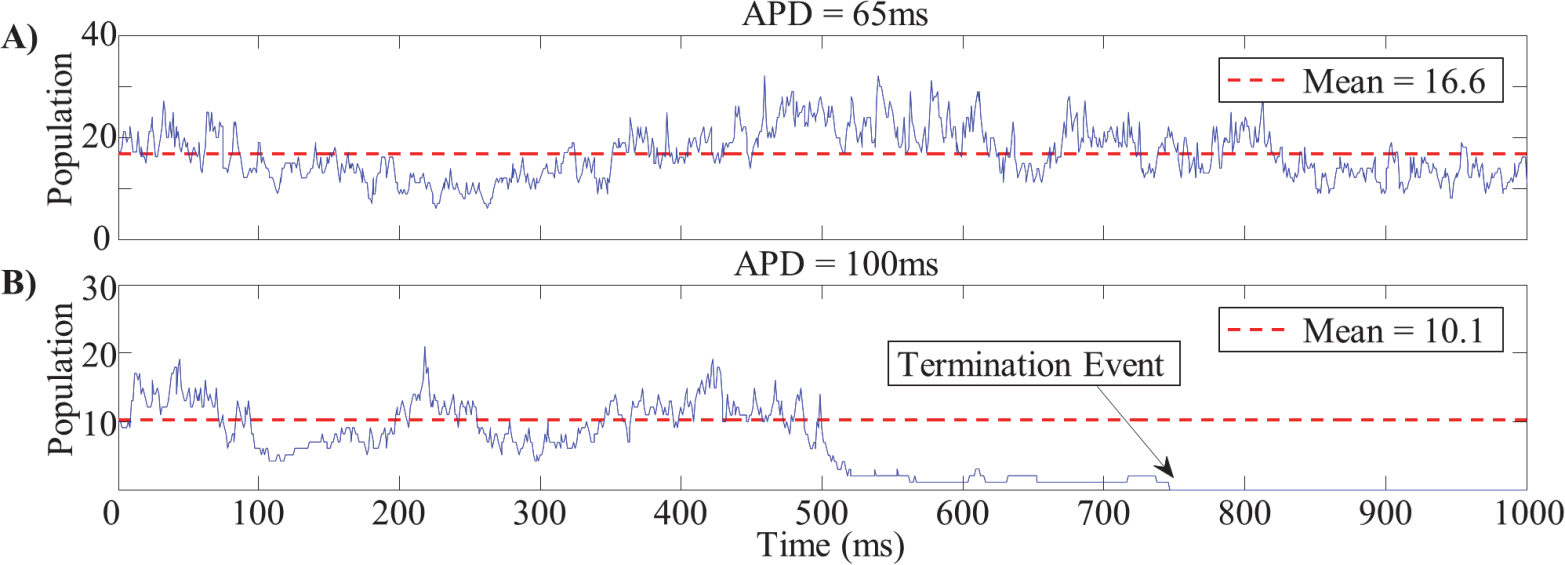
Wave Population Time Series. Examples of wave population time series for short (*A*) and long (*B*) wavelength tissues. The mean wave population for the short wavelength tissue (16.6 waves) was substantially lower than that of the long wavelength tissue (10.1 waves). At approximately 750ms, the wave population in the long wavelength tissue drops to zero and reentry terminates.

At each time step, we located all those cells which had become depolarized within the previous 10ms and defined the individual waves existing at that point in time to be the distinct groups of contiguous depolarized cells (Fig. 1); this allowed us to track the wave population through time. Fig. 2 shows two representative examples of wave population versus time.

We generated three series of rectangular virtual tissues in each of which a single parameter was varied (*A/BL*, *APD* and *RC* as shown in Table 1). Tissue sizes in our model typically ranged between 2,500 and 10,000 cells. This range allows for between 5 and 50 waves to coexist on a tissue depending on that tissue’s properties. This range of wave to chamber size ratios encompasses that found in mapping studies of multi-wavelet reentry in humans [21],[22].

**Table 1:** Tissue Parameter Ranges.

**Tissue Series**	**A/BL**	**APD**	**RC**
**#1 (Varying A/BL)**	12.5 to 25mm	80ms	10ms
**#2 (Varying APD)**	20mm	50 to 100ms	10ms
**#3 (Varying RC)**	20mm	80ms	8 to 16ms
**#4 (Randomized)**	12.5 to 25mm	30ms to 150ms	8 to 16ms

Ranges of model parameter values used in the virtual tissue experiments.

We used a Monte-Carlo approach to determine how both the duration and population dynamics of MWR were affected by the values of these key parameters. In each tissue, we initiated 500 unique episodes of MWR and recorded the durations of each episode up to a maximum of 10,000s or until spontaneous termination occurred. We generated histograms by binning episodes according to their duration to produce probability distributions of episode length. Runs which failed to initiate reentry or lasted the maximum duration were excluded. In those same tissues, we initiated an additional 10 episodes of reentry in which we recorded the activation patterns of each cell for up to a maximum of 10s or until spontaneous termination occurred; these activation patterns were used to calculate the wave population at each time step. Since approximately 2s worth of model time could be produced in 1s of real computing time, individual simulation durations ranged between 1s and 1.5hrs depending on the tissue’s propensity to fibrillate. We generated histograms by binning time steps according to their population level.

We also tested the interaction between multiple simultaneous parameter changes. We generated one hundred additional virtual tissues with randomized properties (Table 1). In each of these tissues we initiated 500 unique episodes of MWR, and recorded the duration of each episode for up to 10,000s or until spontaneous termination occurred.

## Results

**Fig 3:**
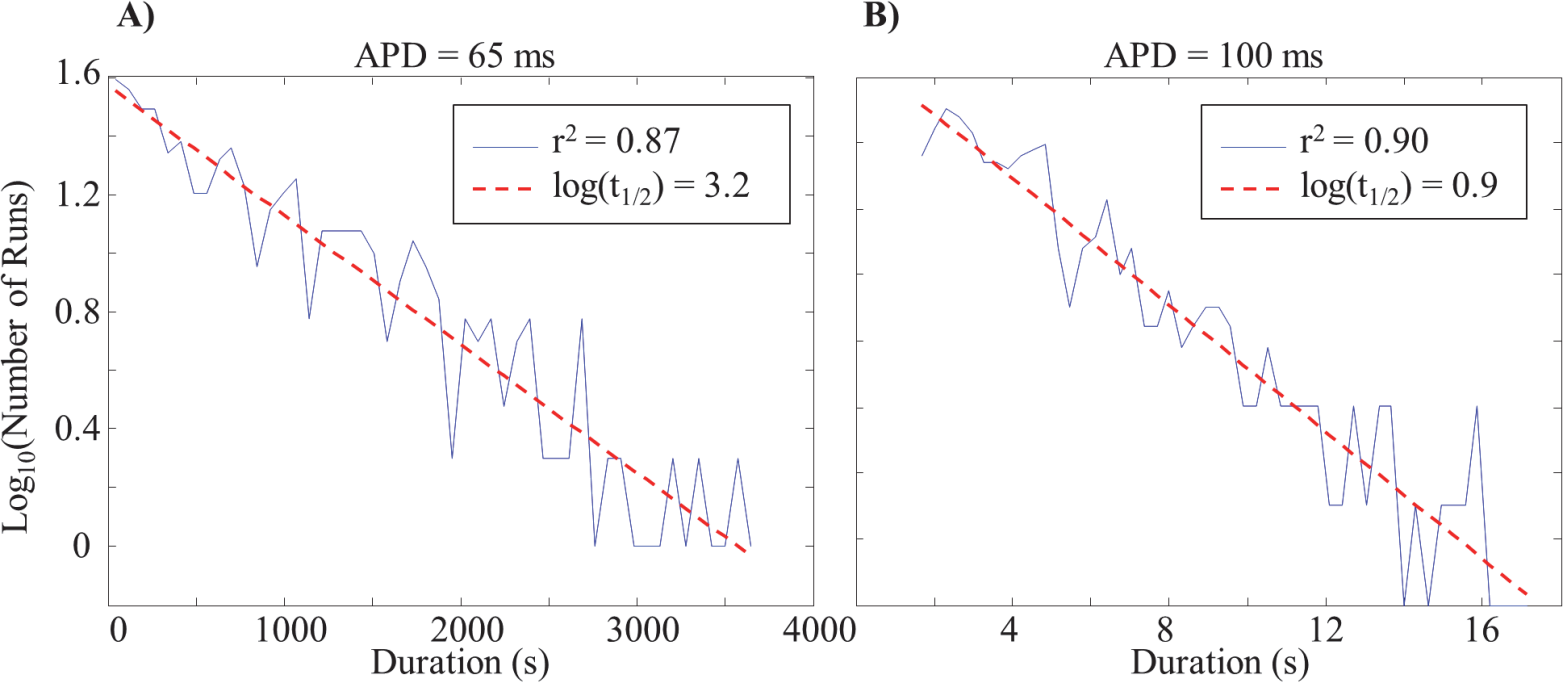
Episode Duration Distributions. Example semi-logarithmic plots demonstrating the exponentially decaying number of MWR episodes to last a given duration. Short wavelength tissue (*A*) had a shallow slope and resulted in a longer half-life (approximately 26 minutes). Long wavelength tissue (*B*) had a steep slope and resulted in a shorter half-life (approximately 7 seconds).

Semi-logarithmic histograms of the number of MWR episodes as a function of duration were well fit by linear regression (Fig. 3), indicating that the number of episodes to last a given duration followed an exponentially decaying distribution. It was therefore possible to calculate the half-life of reentry (*t*_*1/2*_, the amount of time required for half of initiated episodes to terminate) from the slope of these regressions, λ, (Equation 1).

$$t_{1/2}=\frac{\text{ln}\left(2\right)}{\lambda }$$ (1)

**Fig 4:**
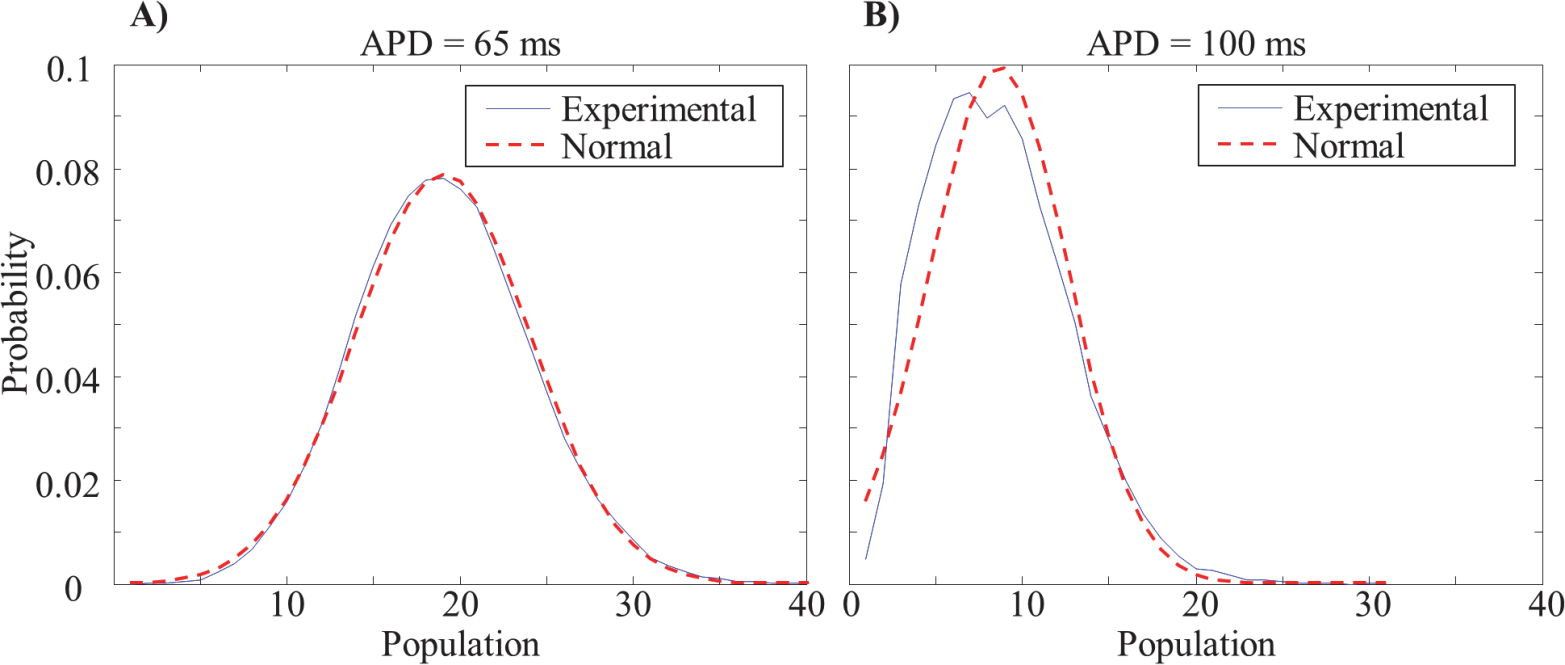
Wave Population Distributions. Probability distributions of the percentage of time spent at each individual wave population for short (*A*) and long (*B*) wavelength tissues (red line—normal distribution).

Normalized histograms of the amount of time spent at a given population level (expressed as relative probability) revealed that the probability of a given number of waves was normally distributed around the mean wave population (mean *r* ^*2*^ = 0.94 over ±2σ from the mean population—Fig. 4).

**Fig 5:**
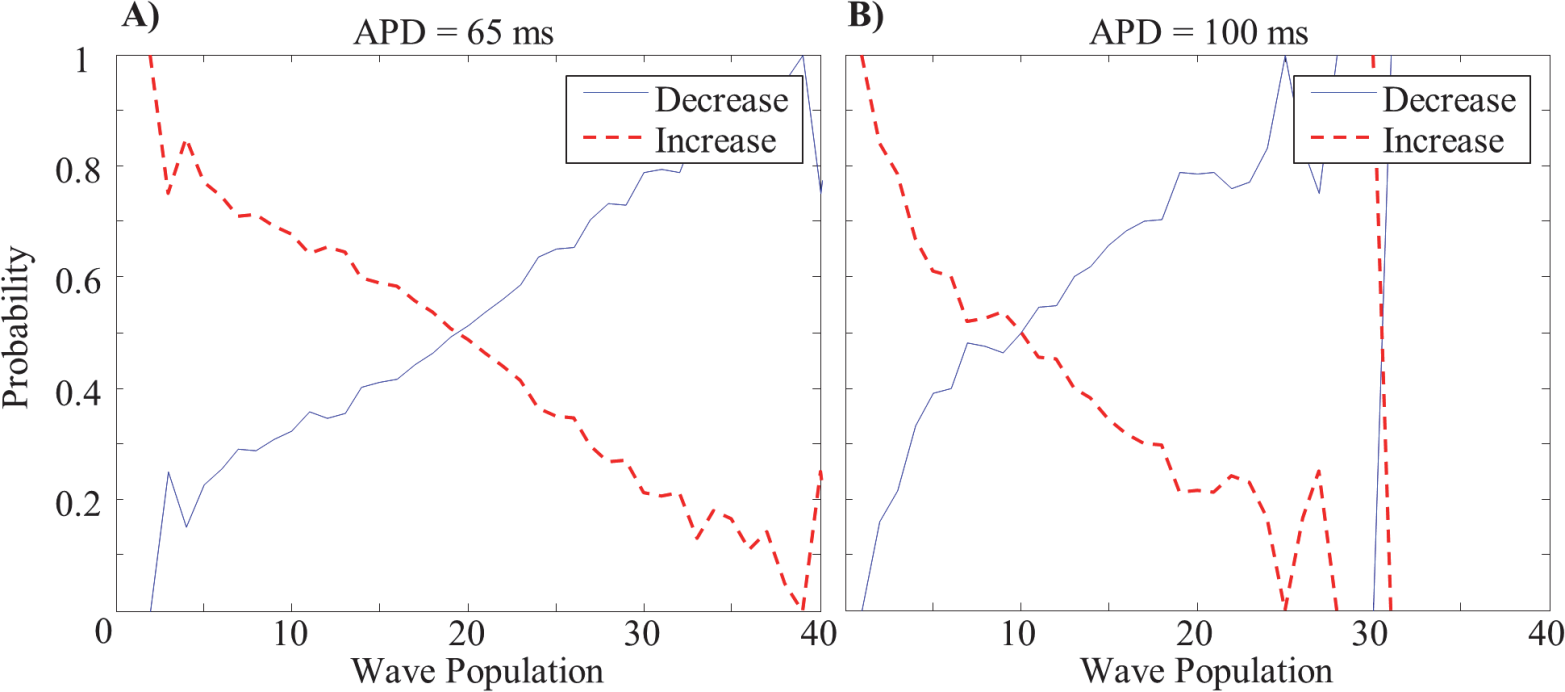
Population Dynamics Probabilities. Representative examples: probabilities of increases or decreases in population as a function of current number of waves. (A) short and (B) long wavelength tissues.

The probability that a population of waves will increase (red) or decrease (blue) in number at the next time step is a function of population size (Fig. 5). When the current population number falls below the mean, the probability that the number of waves will increase at the next time step is increased, and visa-versa. When the population number equals the mean there is an equal probability of having an increase and a decrease in the number of waves at the next time step. The population variance is inversely related to the rate at which these probabilities diverge from one another. The jaggedness of the plots near the highest and lowest populations is due to smaller sample sizes at the population extremes (i.e. fewer naturally occurring instances of these numbers of waves).

**Fig 6:**
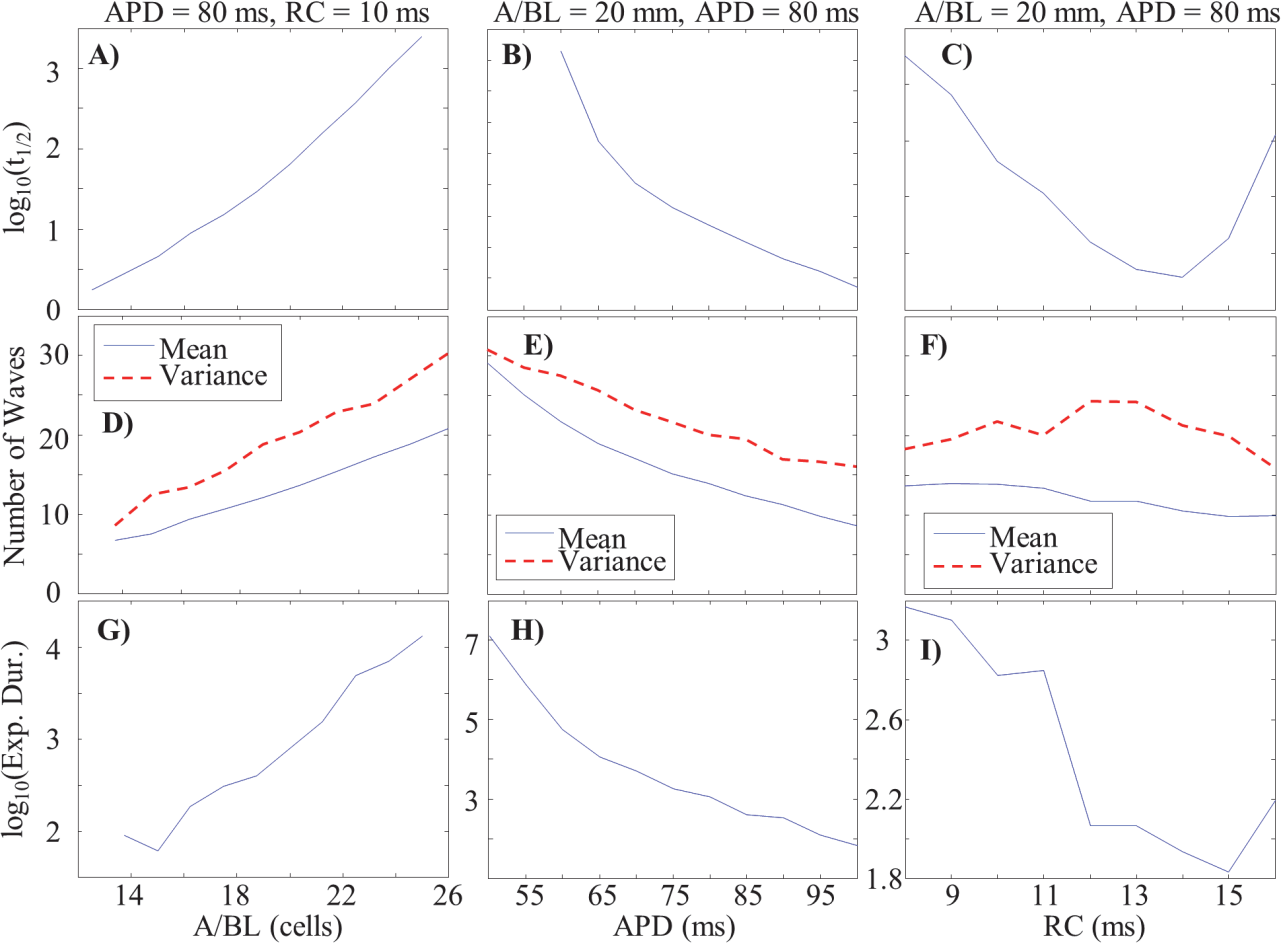
Parameter Dependencies of MWR Behavior. *A-C*: Semi-log plots of MWR half-life as a function of A/BL (*A*), APD (*B*), and RC (*C*). *D-F*: The mean number of waves per time step (shown in blue) and population variance (shown in red) as functions of A/BL (*D*), APD (*E*), and RC (*F*). *G-I*: Semi-log plots of the expected duration of reentry (calculated from the population mean and variance) as a function of A/BL (*G*), APD (*H*), and RC (*I*). Note the similar parameter dependencies of MWR half-life and expected duration.

Half-life increased exponentially with either increasing *A/BL* or decreasing *APD* or *RC* (Fig. 6, A-C). These exponential relationships broke down when the ratio of *RC/APD* reached approximately 0.15–0.20. We were unable to generate values of *t*_*1/2*_ for *APD* values below 60ms due to computational limitations (all episodes lasted until the maximum allowed time, 10,000s).

Mean wave population increased linearly with increasing *A/BL* and decreased linearly with increasing *APD* and *RC* (Fig. 6, D-F). Changes in variance ran largely parallel to changes in population mean for both *A/BL* and *APD* but had a positive dependence on *RC*, increasing until *RC/APD* reached approximately 0.15–0.2 and decreasing at higher values. We were unable to generate meaningful data for *A/BL* values below 13.75 due to the short durations of MWR. We calculated the probability of reaching a zero wave population according to Equation 2 (the cumulative probability below zero of a normal distribution function).

$$P_{\left(\text{population}\leq 0\right)}=\frac{1}{\sigma \sqrt{2\pi }}\int_{-\infty }^{0}e^{-{\left(x-\mu \right)}^{2}/(2\sigma^{2})}dx$$ (2)

Here, μ is the population mean and σ is the standard deviation of the population’s variability. We approximated the expected duration of reentry as the inverse of these cumulative probabilities. For example, if there is a twenty percent chance of termination per second, on average we expect reentry to last 5 seconds. Just as with the dependency of *t*_*1/2*_ on these parameters, increases in expected duration were found with increasing *A/BL*, decreasing *APD*, and decreasing *RC* (Fig. 6, G-I). Similar breakdowns in the linearity of these dependencies were seen at ratios of *RC/APD* of 0.15–0.20.

We composed a metric for a tissue’s propensity to fibrillate that incorporates the effects of what we considered to be the three most relevant parameters (*A/BL*, *APD*, and *RC*) on MWR episode duration. We term this metric the fibrillogenicity index and define it as

$$Fb=\frac{A}{BL*APD*RC}$$ (3)

**Fig 7:**
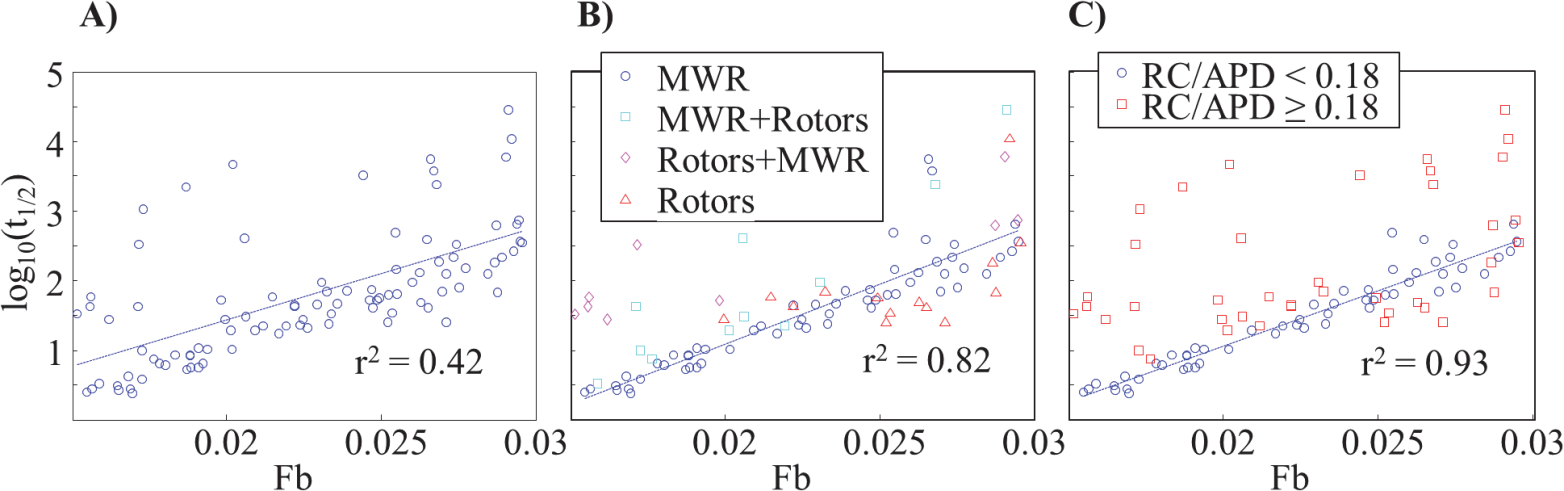
Utility of the Fibrillogenicity Index. The logarithm of the MWR half-lives as a function of the fibrillogenicity index. For all randomly generated tissues, the fit is poor (*A*). Limiting the analysis to tissues displaying only MWR results in a substantially improved correlation coefficient (*B*). Using an RC/APD cutoff of 0.18 to identify likely MWR episodes further improves correlation coefficient (*C*).

**Fig 8:**
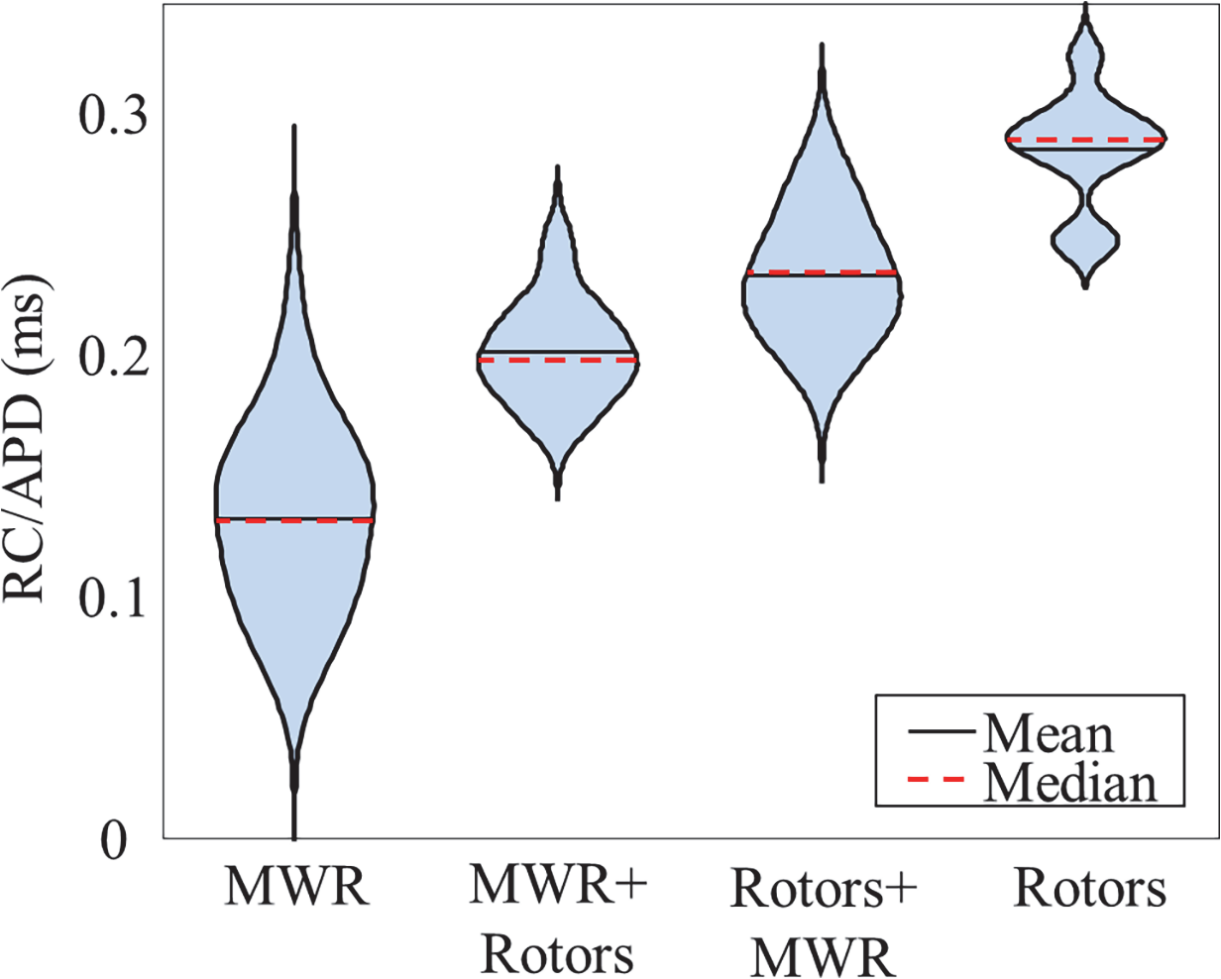
Parameter Dependency of Reentrant Behavior. Violin plot showing the distribution of RC/APD ratios for the four different categories of reentrant behavior. Violin width corresponds to relative sample density.

There was a clear exponential relationship between *t*_*1/2*_ and *Fb*, but an *r* ^*2*^ correlation value of only 0.42 (Fig. 7A). We noticed that the majority of outlying points were exhibiting non-MWR type of behavior. After limiting our plot to tissues displaying only MWR the correlation between log(*t*_*1/2*_) and *Fb* was much greater (*r* ^*2*^ = 0.82, Fig. 7B). We calculated the ratio of *RC/APD* for tissues in each of the four behavior categories (Fig. 8). An analysis-of-variance revealed a statistically significant (*p*<<0.001) difference in this value between groups, suggesting a method for predicting the behavior-type of a tissue based solely on the ratio of *RC/APD*. A receiver-operator curve analysis showed that a *RC/APD* cutoff of 0.18 resulted in a sensitivity of 91.4% with a false positive rate of only 5.7%. Using this cutoff value, we re-plotted log(*t*_*1/2*_) as a function of *Fb* and found that the *r* ^*2*^ correlation coefficient improved to 0.93 (Fig. 7C).

## Discussion

Multi-wavelet reentry can at times appear to be an incomprehensible writhing mass of waves that fuse, divide, and annihilate across the atria in an apparently random fashion. Due to the complex, non-linear dynamics of propagation, it is extremely difficult to predict the long-term destiny of individual waves. However, by analyzing the system’s bulk characteristics rather than the evolving states of each specific wave, we have demonstrated that the ensemble behavior (i.e. MWR duration) can be predicted with a remarkable degree of accuracy. Knowledge of the statistics of wave population dynamics provides the link between atrial properties (size, shape and wavelength) and the duration of MWR episodes. By understanding how and why changes in parameters affect statistical metrics such as population mean and variance, we can predict how those same changes will ultimately alter episode duration.

### Population Dynamics

Changes in wave population result from transient imbalances between wave formation events such as division, and wave reduction events such as fusion or annihilation. These life cycle events are the consequence of specific interactions between waves and their environment: division results from wave-front/wave-tail interactions, fusion results from wave-front/wave-front interactions, and annihilation results from wave-front/boundary interactions. The balance of these processes determines the population mean. The extent to which deviations in the number of waves alter this balance determines how avidly the population returns to the mean. While a reduction in the wave population increases the probability of wave division there is a non-zero probability that the population will continue to decrease and ultimately result in a zero wave population (MWR termination). Lower mean number of waves (which requires fewer sequential reduction events) and higher variance (which reduces the tendency to return to the mean) both result in a higher probability of reaching zero population and termination.

### Tissue Properties, Wave Population and MWR Duration

By influencing the relative probabilities of wave division, fusion and annihilation, tissue properties determine the duration of MWR episodes. Increasing the ratio of tissue area to boundary length (*A/BL*) decreases the rate of wave annihilation relative to wave fusion and division and thus increases both mean number of waves and MWR duration. Increasing *APD* increases wavelength and therefore the area encompassed by individual waves. This preferentially increases the likelihood of annihilation and fusion and leads to a decrease in both the mean number of waves and MWR duration. As *RC* increases wavelength decreases but there is a concomitant increase in the excitable gap between waves. The combined effect is to gradually decrease mean number of waves and increase population variance, and ultimately leads to a decrease in MWR duration. Sufficient increase in the excitable gap reduces wave-wave interactions and allows for the formation of stable rotors.

The qualitative relationship between a tissue’s properties (area, boundary length and wavelength) and its capacity to maintain fibrillation has been studied in both animal models and humans. Garrey was the first to formulate the ‘mass hypothesis’, demonstrating that the duration of fibrillation correlated with tissue area and width (no measurements of wavelength or refractoriness were performed) [11]. Subsequently the role of wavelength (as well as tissue size) was shown to correlate with AF duration in animal models [23],[24] and in humans [25]. While it has long been recognized that increases in A/BL or decreases in APD will result in increased duration of multi-wavelet reentry [17],[25],[24], this study presents the first quantitative analysis of these dependencies (e.g., linear vs. exponential, etc.).

The fibrillogenicity index captures the balance between the two major forces which affect wave population dynamics: the size and shape of the tissue in which MWR exists (as determined by *A/BL*), and the size and shape of the waves themselves (as determined by *APD* and *RC*). So long as these opposing forces are scaled together, the absolute values of individual parameters are inconsequential. As an important caveat, our probabilistic formulation for predicting the duration of MWR is dependent upon individual waves having a non-zero probability of interacting with other waves or with tissue boundaries. Accordingly, we found that as pure MWR transitions to include spatially stable rotors, the fibrillogenicity index becomes less predictive of episode duration.

### Application of the Fibrillogenicity index to Therapy

Perhaps the most promising aspect of the fibrillogenicity index is its potential application in treatment strategies. A common approach in deciding which lesion set to apply is to classify patients as having either paroxysmal (< 7 days) or persistent (≥7 days) AF [7]. Ablation is typically limited to pulmonary vein encirclement and isolation in paroxysmal AF, whereas patients with persistent AF are felt to require further atrial substrate modification (such as roof and isthmus lines or ablation of complex fractionated atrial electrograms (CFAE) [4],[8]). While this binary approach can be effective in patients whose AF durations are less than a few months, cure rates are 50% or lower in patients with longer lasting AF [6]. Obviously, standard treatment protocols are inadequate within this population. Regardless of which strategy one chooses for deciding where to ablate, a method is required for determining *how much* to ablate. In other words, if more extensive ablation is called for, by what degree should it be increased? Optimization of any approach to targeted ablation will require a titration method for determining the extent of lesions necessary at driver sites. The fibrillogenicity index has been designed to provide just such guidance, and thus offers a potentially useful compliment to any map-guided ablation strategy.

### Limitations

As in any purely *in silico* experiment, the generalizability of our results remains limited and these findings will require validation in a biologic system, ultimately humans. None the less, computational modeling does offer some unique and powerful advantages over biologic experimental preparations. Only through the use of a computational model was it possible to explicitly isolate and manipulate each variable independently. Furthermore, the Monte-Carlo approach we made use of in characterizing probability distributions of MWR episode durations relied on collecting data from a large number of reentrant episodes. This type of high volume data collection is not possible in the clinical arena. Even within the computational arena however, we restricted our analysis to highly idealized instances of atrial substrate (two dimensional rectangular tissues with homogeneously distributed cell properties). Future extension of the fibrillogenicity index must account for the complex geometries, regional heterogeneities and anisotropy found in human hearts.

Our probabilistic formulation of wave population dynamics applies only to situations in which the positions of reentrant circuits change chaotically. However, as intercellular resistance and action potential duration are altered to produce a shortened propagation wavelength and a smaller radius of curvature, multi-wavelet reentry resolves gradually into a more spatially stable, rotor-driven, form of reentry. The phenotype of reentrant behavior is dependent on the ratio of RC/APD. As with the magnitudes of other parameters presented in this study, a direct mapping to the biological realm would be inappropriate. Instead, it is the way in which a relative change in these parameters affects the ultimate behavior of the tissue which is of interest. For example, any decrease in APD increases not only the duration of reentry, but also the likelihood of rotor-driven reentrant forms.

We have not explicitly addressed the impact of remodeling in our simulations. It has been demonstrated that progressive changes to ion channel expression and development of interstitial fibrosis can increase AF burden [26]. For example, coupling between myocytes and fibroblasts/myofibroblasts has been shown to alter APD, resting membrane potential, and conduction velocity [27],[28], and can ultimately lead to an increase in tissue’s propensity to fibrillate [29]. Remodeling effectively describes the movement through parameter space that results from feedback between behaviors (AF itself) and parameters (e.g. resistance, capacitance, APD). By focusing only on the steady state situation we have defined the mapping between model parameters and behavior (i.e. Fb). Thus with the fibrillogenicity index we can predict the impact, though not the dynamics, of remodeling.

The fibrillogenicity index is calculated from parameters that we have complete knowledge of in the computational environment but which are difficult to measure in the clinical setting. Though it is relatively straightforward to gather information regarding the size and shape of a patient’s atria (e.g. via CT scan or electro-anatomic mapping), measurements of APD, resistance and capacitance cannot easily be obtained. We have previously demonstrated that electrogram frequency (when acquired using electrodes with high spatial resolution) correlates well with tissue wavelength and may therefore offer a practical, measurable alternative for calculating the fibrillogenicity index.

## Conclusions

We report a mechanistic, population dynamics based framework for understanding multi-wavelet reentry using a computational model of propagation. During MWR, the number of waves which coexist on a given tissue follows a normal distribution, the mean and variance of which can be used to calculate the probability of spontaneous termination. Three key parameters influence the duration of reentrant episodes: tissue size and shape, action potential duration, and resistance and capacitance. Increases in the tissue-area to boundary-length ratio, or decreases in *APD* or *RC* resulted in exponential increases in the duration of MWR episodes. We demonstrated that the phenotype of reentrant behavior (MWR vs. spatially stable rotors) is dependent upon the ratio of *RC/APD*; below a ratio of 0.18 reentry reliably fits into the category of pure MWR. Finally, we have defined a metric for quantifying a tissue’s propensity to fibrillate. The fibrillogenicity index is derived from a tissue’s properties and accurately predicted MWR half-life over a wide range or durations (1s to 6hrs, *r* ^*2*^ = 0.93). We believe that the fibrillogenicity index provides a foundation upon which patient-specific treatment protocols for AF can be built.

## Supporting Information

S1 Video Categories of Reentry. In this video, we present examples from four different positions along the spectrum of reentrant behavior: 1) pure MWR, 2) MWR with occasional rotor formation, 3) rotors with occasional bouts of MWR, and 4) stable rotors.
(MP4)

S1 Table Complete Results Data Set. (XLSX)
